# Evaluation of Novel Glass Fiber-reinforced Composite Technique for Primary Anterior Teeth with Deep Carious Lesions: A 12-month Clinical Study

**DOI:** 10.5005/jp-journals-10005-1421

**Published:** 2017-06-01

**Authors:** Ajinkya Sawant, Yusuf Chunawalla, Abdul Morawala, Nupur S Kanchan, Kapil Jain, Rohan Talathi

**Affiliations:** 1 Private Practitioner, White On Whites Dental Care, Mumbai, Maharashtra, India; 2 Professor and Head, Department of Pedodontics and Preventive Dentistry, M. A. Rangoonwala College of Dental Sciences & Research Centre Pune, Maharashtra, India; 3 Senior Lecturer, Department of Pedodontics and Preventive Dentistry, M. A. Rangoonwala College of Dental Sciences & Research Centre Pune, Maharashtra, India; 4 Private Practitioner, Daffodills Pediatric Dental Home, Pune, Maharashtra, India; 5 Postgraduate Student, Department of Pedodontics and Preventive Dentistry, M. A. Rangoonwala College of Dental Sciences & Research Centre Pune, Maharashtra, India; 6 Senior Lecturer, Department of Pedodontics and Preventive Dentistry, M. A. Rangoonwala College of Dental Sciences & Research Centre Pune, Maharashtra, India

**Keywords:** Anterior esthetic restorations, EverStick glass fiber-reinforced composite post, ParaPost Taper Lux post, Primary maxillary incisors.

## Abstract

**Background:**

Early childhood caries discloses a distinct clinical pattern, and the teeth most often involved are the maxillary central incisors, lateral incisors, and the maxillary and man-dibular first molars. The maxillary incisors are most severely affected, with deep carious lesions usually involving the pulp. Teeth that have been endodontically treated often have little coronal tooth tissue remaining and as such require a post to retain the core and restoration .This study evaluated and compared the efficacy of EverStick glass fiber-reinforced composite post with ParaPost Taper Lux in primary maxillary anterior teeth.

**Aim:**

An *in vivo* study was conducted to evaluate and compare the longevity and failures of two fiber post systems in primary maxillary anterior teeth.

**Materials and methods:**

A total of 60 severely mutilated primary maxillary anterior teeth from children aged 3 to 5 years were selected according to the inclusion criteria. These teeth were treated endodontically and were randomly assigned into two groups with 30 samples in each group, group I: EverStick glass fiber-reinforced composite post, group II: ParaPost Taper Lux post. The evaluation of dislodgment of posts, secondary caries, root fracture, and post fracture was carried out clinically and radiographically during every follow-up at 3, 6, 9, and 12 months interval.

**Results:**

Statistical tests (Chi-square test, Fisher’s exact probability test) suggested that dislodgment of the posts was significant between the two groups at 6, 9, and 12 months follow-ups. But within the group during subsequent follow-up intervals, dislodgment of posts as a mode of failure was not statistically significant. However, clinically failures were seen in both the study groups.

**Conclusion:**

Fiber post system has proved to be successful clinically in both primary and permanent teeth due to the mono-block effect with luting agent, post system, core material, and bonding to dentin. Thus, today the EverStick glass fiber post system provides a novel way of fabricating cost-effective and less time-consuming custom-made post in treating mutilated maxillary anteriors.

**How to cite this article:**

Sawant A, Chunawalla Y, Morawala A, Kanchan NS, Jain K, Talathi R. Evaluation of Novel Glass Fiber-reinforced Composite Technique for Primary Anterior Teeth with Deep Carious Lesions: A 12-month Clinical Study. Int J Clin Pediatr Dent 2017;10(2):126-130.

## INTRODUCTION

Despite the fact that it is largely preventable, dental caries is the most common chronic disease of childhood. Caries in very young children known as early childhood caries may be defined according to the American Academy of Pediatric Dentistry “as the presence of one or more decayed, missing (due to caries) or filled tooth surfaces in any primary tooth in a child 71 months of age or younger.“^1^

Early childhood caries discloses a distinct clinical pattern, and the teeth most often involved are the maxillary central incisors, lateral incisors, and the maxillary and mandibular first molars. The maxillary incisors are most severely affected, with deep carious lesions usually involving the pulp. In extreme cases, early childhood caries can even lead to loss of the crown structure.^2^ If left untreated, health as well as esthetics may be compromised.^3^ The implications of this situation include insufficient growth and development in children who have no other medical problems.^4^ Decreased dietary intake may develop into nutritional imbalance, which may affect the general status of health as well as dentition.^5^

Teeth that have been endodontically treated often have little coronal tooth tissue remaining and as such require a post to retain the core and restoration. There are several types of root canal posts available for use in pediatric restorative dentistry, including prefabricated, orthodontic wire in α or Ω forms, metallic posts with macroretention, short posts with composite resin, polyethylene ribbon posts, and biologic posts.^6^ Fiber-reinforced composite posts was introduced to dentistry around 15 years ago, and they are composed of glass quartz and carbon fibers embedded in epoxy resin. This study evaluated and compared the efficacy of EverStick glass fiber-reinforced composite post with ParaPost Taper Lux in primary maxillary anterior teeth.

## MATERIALS AND METHODS

A randomized controlled single blind *in vivo* study was carried out in which a total of 60 severely mutilated primary maxillary incisors were selected from children aged 3 to 5 years based on the inclusion criteria such as:

- Children between 3 and 5 years
- Mentally and physically normal child with no systemic manifestations
- Pulpally involved maxillary deciduous anterior teeth
- Teeth with more than two-thirds root length
- At least 1 mm of supragingival noncarious tooth structure remaining

Exclusion criteria:

- Mentally or physically compromised child
- Presence of deleterious oral habits
- Presence of trauma from occlusion
- Teeth with pathologic root resorption
- Teeth with presence of root caries
- Teeth with hyoplasia

**Fig. 1:**
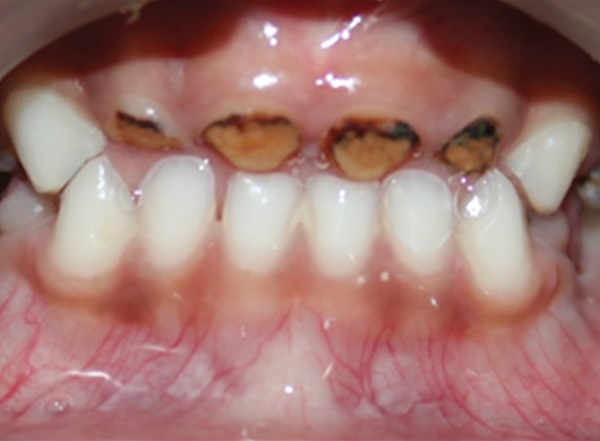
Preoperative intraoral view

**Fig. 2:**
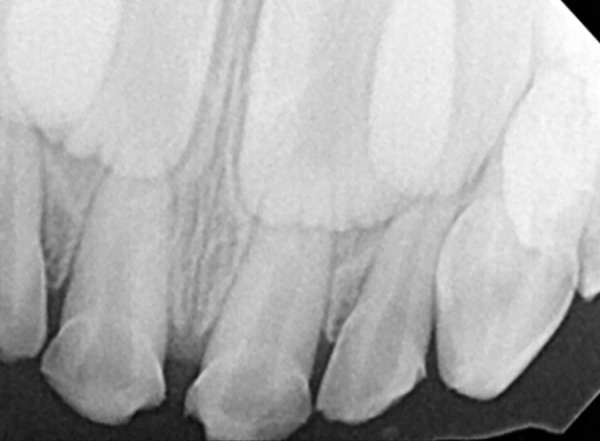
Preoperative radiograph

**Fig. 3:**
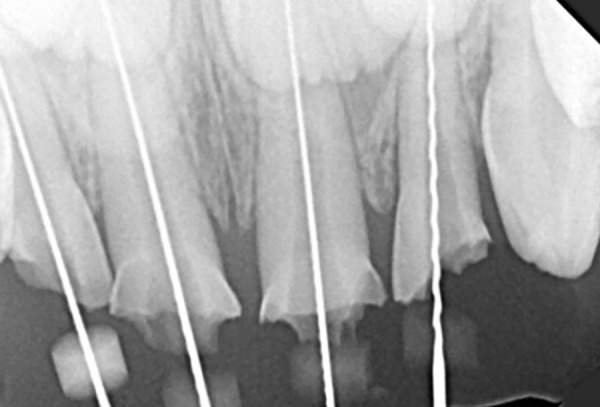
Working length determination

**Fig. 4:**
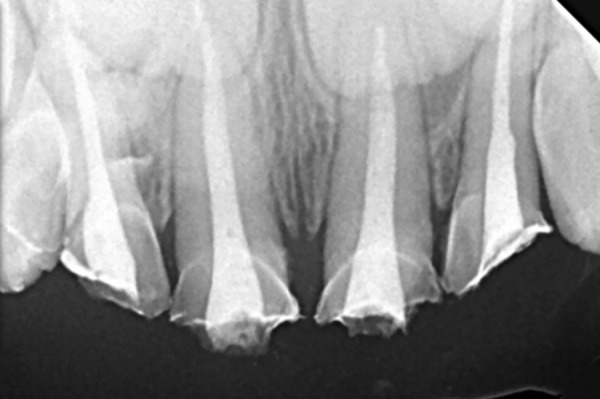
Obturation

**Figs 5A and B:**
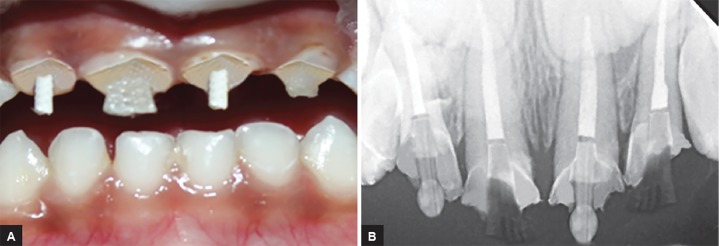
(A) Post placement clinically; and (B) radiographically

**Figs 6A and B:**
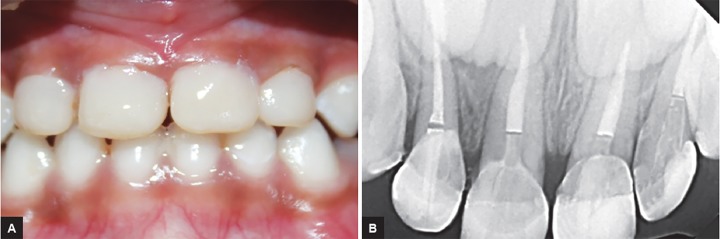
Postoperative intraoral view and radiograph showing restored primary maxillary anterior teeth

These selected teeth were endodontically treated and were further divided into two groups: Group I (EverStick glass fiber-reinforced composite post) and group II (Para-Post Taper Lux post) with 30 samples in each group. The above-mentioned procedure is described in Figures 1 to 6. The evaluation of dislodgment of posts, secondary caries, root fracture, and post fracture was done clinically and radiographically during every follow-up at 3, 6, 9, and 12 months interval.

## RESULTS

**Graph 1:**
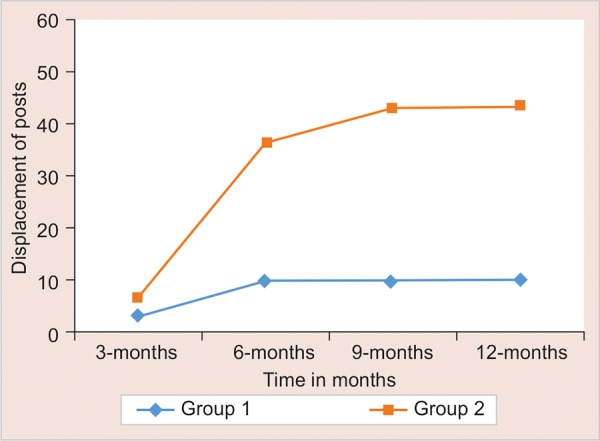
The distribution of displacement of posts between two groups

**Graph 2:**
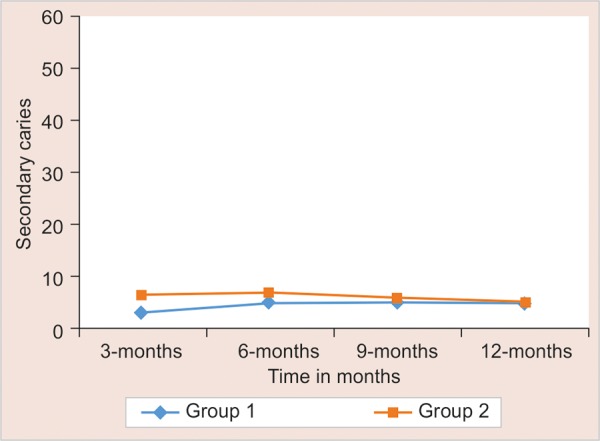
Secondary caries

**Graph 3:**
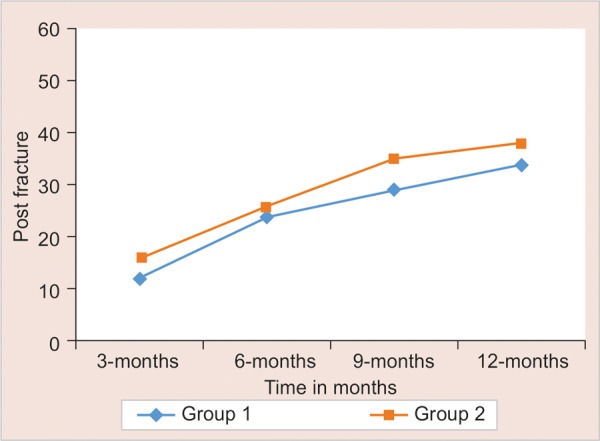
Post fracture

**Graph 4:**
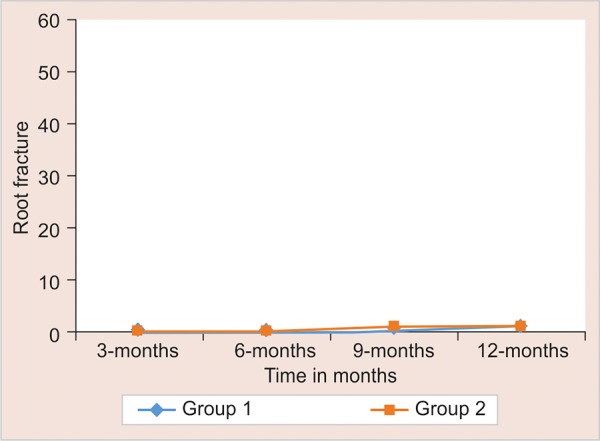
Root fracture

The evaluation of dislodgment of posts, secondary caries, root fracture and post fracture was done during every follow-up at 3, 6, 9, and 12 months interval. Statistical significance of difference of each outcome measure is tested using Chi-square test for independence of attributes if cell frequency is larger than 5; else Fisher’s exact probability test was applied. The results are mentioned in Graphs 1 to 4.

## DISCUSSION

In early childhood caries, maxillary anterior teeth are the most affected, mainly because they erupt before molars and are exposed to carious environment for longer duration; they are not protected by tongue, as are the mandibular incisors and they do not have close proximity to salivary gland duct openings. This is in accordance with the progression pattern of early childhood caries stated by Ripa.^7^

Carious lesions presented in maxillary anterior teeth are associated with problems like reduced vertical dimension, masticatory insufficiency, esthetics, development of parafunctional habits, and psychological problems. Hence, it is necessary to restore and preserve them.^8^ Problems with anterior primary incisor teeth that are grossly decayed are the lack of coronal structure to support and provide adhesion for a composite resin. In such cases, the use of an intracanal post in endodontically treated teeth improves the retention for a longer-lasting restoration. Rifkin described restoring primary anterior teeth with post and crown. But it was not widely accepted because of the potential for interference with physiologic root resorption if the wire extends a long way into the root.^9^

The development of fiber-reinforced composite technology has brought a new material into the realm of metal-free adhesive esthetic dentistry.^10^ Glass fiber-reinforced composite posts (EverStick, Turku, Finland) are esthetic and easy to use. Flexural strength of silanated E-glass fiber (EverStick) is 1,280 MPa, highest among all fiber-reinforced composite posts. The modulus of elasticity is close to dentin, which helps in even distribution of stress.^10^ These posts are marketed custom-made posts. These can be manipulated by the operator according to the requirements. The length, width, taper of the post can be manipulated by the operator according to needs. Success of a post also depends on the fit of the post into the canal. Preformed posts may not snugly fit into the canal. Fiber posts (EverStick) can be modified for a snug fit.^11^ Additional fibers can also be inserted in cases of wide canals.

Occurrence of root fracture was not seen in any of the cases (100% success) during the 12 months follow-up period (comparing groups I and II, p = 0.999). Baraju et al in their study found that the modulus of elasticity of the fiber post is close to dentin and hence, there is less stress distribution toward the dentinal walls, making the root less susceptible to fracture.

It was noted that most of the restorations adapted closely to tooth along margins. There was no gap between restoration and tooth interface. This is attributed to the absence of secondary caries (comparing groups I and II, p = 0.999). None of the 60 cases reported with secondary caries (100% success).

In this study, at the 3-month recall, 1 out of 30 cases in group I and II out of 30 cases in group II showed failure due to dislodgment of the posts. At 6-month recall, 3 out of 30 cases in groups I and 11 out of 30 cases in group II showed failure due to dislodgment of the posts. The difference between the groups was statistically significant. EverStick glass fiber composite is a custom-made post providing ease of manipulation and snug fit in the canal.^12^ No additional post space preparation is required for the insertion of the post. ParaPost Taper Lux is a prefabricated post requiring additional post space preparation. Preparation of a post space requires removal of additional radicular dentin beyond the requirements for root canal treatment.^13-15^ Thus, the strength of the post is derived from interface between luting agent and the post, resulting in dislodgment of the post.

## CONCLUSION

In agreement with previous studies noted in the litera-ture,^11,12^ for rehabilitation of extensively decayed primary incisors, the use of EverStick glass fiber-reinforced composite posts appears to be a cost-effective alternative option, in view of their ability to reinforce composite resin with adequate translucency, durability, improving esthetics, retention, and marginal adaptation.^16,17^
